# Complicated Type B aortic dissection following Type A aortic Dissection Repair: Case Report

**DOI:** 10.1186/1749-8090-10-S1-A90

**Published:** 2015-12-16

**Authors:** Isabel Pereira, John Quarmby

**Affiliations:** 1 Royal Derby Teaching Hospital, Uttoxeter Road, Derby DE22 3NE, UK

## Background/Introduction

Aortic dissection is an uncommon surgical emergency it is most lethal disease of the aorta. Prompt diagnosis and management of aortic dissection are key to reduce patient morbidity and mortality; hence the need to a have a high index of suspicion for this condition.

## Method

A 47 year old gentleman presented with chest pain radiating to the back which started during coitus. On admission he was diagnosed with hypertensive crisis and was given a GTN infusion. A CT scan of the aorta showed full length aortic dissection including the brachiocephalic trunk and both common iliac arteries. He was transferred to the Cardiac Unit for surgical repair of the type A aortic dissection, with replacement of the aortic valve and ascending aorta with inter-positional tube graft for type A dissection. Five days post operatively he re-presented with chest, back and abdominal pain associated with diarrhoea. He required Intensive Care Unit support. The patient then had an urgent CT Angiogram which showed progression of the dissection, involving type B aortic dissection extending to both common iliac arteries with significant stenoses of the true lumen of the aorta and right common iliac artery. Clinically there was evidence of bowel ischaemia and right lower limb ischaemia. He had prompt surgical fenestration of the abdominal aorta, retrograde recanalization, perfusion of the superior mesenteric artery, and embolectomy of the right common iliac artery. A short segment of the small bowel was ischaemic with petechial haemorrhages. Twenty-four hours after restoring vascular supply a re-look laparotomy confirmed that the bowel was viable. Post operatively he made an uneventful recovery, follow up CT scans have not revealed any new changes to the aortic dissection.

## Discussion/Conclusion

This case illustrates, that following repair of type A aortic dissection the dissecting flap may continue to propagate distally and become unstable. This may have deleterious effects to end organs if surgical action is not taken promptly. Patients should never be profiled, and chronic complaints should always be revisited.

## Consent

Written informed consent was obtained from the patient for publication of this abstract and any accompanying images. A copy of the written consent is available for review by the Editor of this journal.

